# Supplementary material for: Atrial scar quantification via multi-scale CNN in the graph-cuts framework
Source: Med Image Anal. 2020 Feb;60:101595. doi: 10.1016/j.media.2019.101595 (PMC6988106; doi:10.1016/j.media.2019.101595)
Supplement: Supplementary Data S1 — Supplementary Raw Research Data. This is open data under the CC BY license http://creativecommons.org/licenses/by/4.0/ [file mmc1.pdf]

Supplementary Material Document:
Atrial Scar Quantification via Multi-scale CNN in the Graph-cuts Framework

Lei Li[a,b], Fuping Wu[c], Guang Yang[d,e], Lingchao Xu[f], Tom Wong[e], Raad Mohiaddin[d,e], David Firmin[d,e], Jennifer Keegan[d,e], Xiahai Zhuang*[b]

[a] *School of Biomedical Engineering, Shanghai Jiao Tong University, Shanghai, China*
[b] *School of Data Science, Fudan University, Shanghai, China*
[c] *Dept of Statistics, School of Management, Fudan University, Shanghai, China*
[d] *National Heart and Lung Institute, Imperial College London, London, UK*
[e] *Cardiovascular Research Center, Royal Brompton Hospital, London, UK*
[f] *School of NAOCE, Shanghai Jiao Tong University, Shanghai, China*

1. Cube-shaped patch experiment

We performed an experiment to evaluate the performance of the proposed LearnGC algorithm with different patch shapes and sizes, including four cube-shaped patches and six elongated shape patches. The four cube-shaped patches were with sizes of $11 \times 11 \times 11$, $13 \times 13 \times 13$, $15 \times 15 \times 15$, and $17 \times 17 \times 17$ pixel; the sizes of the six elongated shape patches ranged from $7 \times 7 \times 11$ to $17 \times 17 \times 21$ pixel. The pixel size was $1 \times 1 \times 1$ mm. The balancing parameter λ of the graph-cuts was set to 0.6, and the random shift range $\mathcal{R}$ was set to half of the patch length.

As Fig. 1 shows, the average Dice scores of the cube-shaped patches were not better than those of the elongated patches, which have smaller volumes and are computationally more efficient. It may be due to the fact that the texture information along the perpendicular direction to LA boundaries/ surfaces is more important in the classification of scars. Particularly, this elongated shape patches can mitigate the errors caused from the inaccurate delineation of the LA boundaries. Additionally, it can be less efficient in time and memory to use the cube-shaped patches to include the same amount of texture information along different directions. In summary, the elongated shape patches are more suitable for our task compared to the cube-shaped patches.

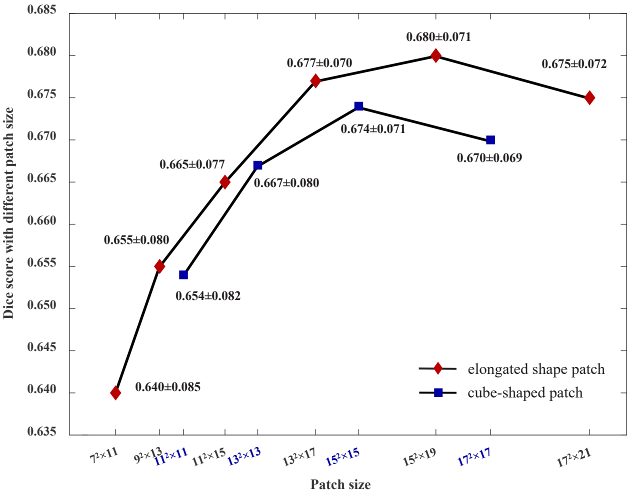

Figure 1: Dice scores of the proposed method with different shapes and sizes of patches.

2. An end-to-end graph learning test

The lack of end-to-end training could limit the proposed segmentation pipeline to fully benefit from the n-link strategy, and an end-to-end graph learning network can be helpful. Here, we implement an end-to-end training network for the scar segmentation. To clarify the designation of the network, we first drew a mind map, presented in Fig. 2, and elaborate on the idea in Section 2.1. Section 2.2 presents the proposed conditional random field (CRF) deep neural network. We studied the effect of different numbers of edges and patch sizes in Section 2.3. Finally, Section 2.4 complementally discusses the function of the n-link weight.

2.1. The mind map of an end-to-end graph learning network

The mind map of implementing an end-to-end learning graph network is presented in Fig. 2. To extract the features for classification of a graph node, one could use either the intensity value of the image at the corresponding coordinate of the graph node, or an image patch centered at the coordinate of the graph node. The former is referred to as the *pixel-based classification*, and the latter is referred to as the *patch-based classification*. In the patch-based classification, training the network based on the whole graph could be too memory-expensive to implement; alternatively, one could implement them using subgraphs extracted from the whole graph. Below we discuss

URL: `zxh@fudan.edu.cn` (Xiahai Zhuang*)

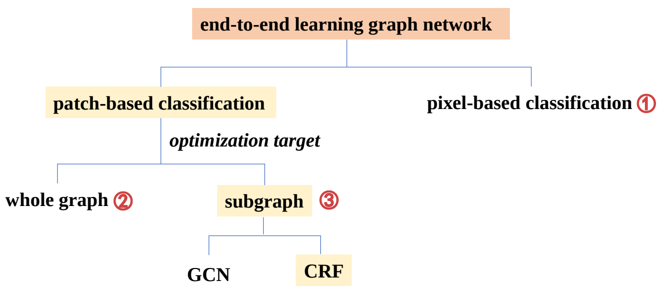

Figure 2: The mind map of an end-to-end graph network.

the three situations labeled in Fig. 2.

(1) The pixel-based classification only includes very limited information, i.e., the intensity value of one pixel, for the clarification of the graph nodes. Hence, it is not applicable in our surface-based segmentation of the LA scars, because the inaccurate delineation of the LA surfaces can lead to wrong intensity values.

(2) The average number of the target mesh nodes and edges are about *2.87e+04* and *1.70e+05*, respectively. Owing to the weighted sampling strategy, the average number of the mesh nodes extracted in the training phase is about *1.45e+04*. Moreover, the element in the proposed framework is not a single pixel, but a patch, e.g. with a size of $13 \times 13 \times 17$ pixel. Hence, the required GPU memory of every single node is about *297.3 MB* for single-scale T-NET and *483.3 MB* for single-scale N-NET. As a result, the average memory required for training an end-to-end LearnGC network with whole graphs could reach over *29,745 GB* for one training case. This is infeasible to implement on a state-of-the-art GPU workstation. Note that the down-sampling scheme should not be applied to the graph to reduce memory requirement, because there could be small scars missed by the down-sampling operation.

(3) To implement the patch-based classification with acceptable computation complexity, we propose to train a network using subgraphs extracted from the whole ones. In this implementation, the label distribution of pixels in different subgraphs are assumed to be conditionally independent. With this assumption, we could design a CRF-based deep neural network, referred to as CRF-Net, for the classification of graph nodes in an end-to-end fashion.

2.2. *CRF-Net: conditional random field deep neural network*

2.2.1. *Formulation*

Let $\mathbf{X}$ be a random field defined by a set of variables $\{x_1, ..., x_n\}$, where x_i is the label assignment for node i. The domain of each variable is a set of labels $\mathbf{L} = \{l^{scar}, l^{myo}\}$ in our work. $\mathbf{I}$ is the target graph surface with n nodes, and I_i is the intensity of node i. CRF meets the

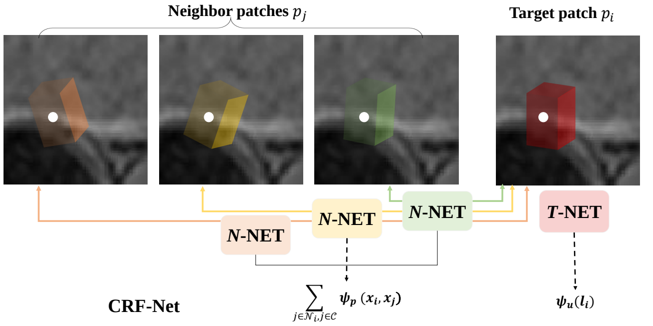

Figure 3: The CRF-Net framework. Note that the diagram takes three edges for each node as an example.

Gibbs distribution, $P(\mathbf{X}|\mathbf{I}) = \frac{1}{Z} \exp(-\sum_{\mathcal{C} \in \mathcal{G}} E(\mathbf{X}_{\mathcal{C}}|\mathbf{I}))$, where energy functions E can be defined as follows:

$$E(\mathbf{X}_{\mathcal{C}}) = \sum_{i \in \mathcal{C}, \mathcal{C} \in \mathcal{G}} \psi_u(x_i) + \sum_{j \in \mathcal{N}_i, j \in \mathcal{C}} \psi_p(x_i, x_j), \quad (1)$$

where $\mathcal{C}$ is the subgraph from the whole graph $\mathcal{G} = (\mathcal{X}, \mathcal{E})$, and $j \in \mathcal{N}_i$ donates a center node i and its neighboring nodes j. In conventional CRF (Krähenbühl and Koltun, 2011), the unary potential $\psi_u(x_i)$ is computed independently for each patch, and the pairwise potentials $\psi_p(x_i, x_j)$ have the form:

$$\psi_p(x_i, x_j) = \mu(x_i, x_j)(w^{(1)} \exp(-\frac{d_{ij}^2}{2\sigma_\alpha^2} - \frac{|I_i - I_j|^2}{2\sigma_\beta^2}) + w^{(2)} \exp(-\frac{d_{ij}^2}{2\sigma_\gamma^2})), \quad (2)$$

where μ is a label compatibility function, d_{ij} is the distance between the neighbor patches p_i and p_j.

In this work, we propose to directly learn and predict these two weights, i.e., $\psi_u(x_i)$ and $\psi_p(x_i, x_j)$, using convolutional network in an end-to-end fasion.

Table 1: Summary of the quantitative evaluation results with the following experiment setup: patch size=$7 \times 7 \times 11$ pixel, No. of edges =3, $\lambda = 0.6$, N_s=1.

Method	CRF-Net	T-NET$^{\#}$	LearnGC$^{\#}$
Dice	0.598 ± 0.078	0.593 ± 0.082	0.640 ± 0.085

2.2.2. *Architecture*

As Fig. 3 shows, the $\psi_u(x_i)$ and $\psi_p(x_i, x_j)$ can be directly predicted using the T-NET and N-NET. Based on the predicted weights, we can obtain the label probability, $\hat{L}$, using Gibbs distribution, *where the normalization term Z is approximatively calculated only considering the target node for simplification.* Hence, the loss function of the proposed CRF-Net can be parameterized by $\theta_{\mathcal{C}}$,

$$\mathcal{L}_{\mathcal{C}}(l_{\mathcal{C}}; \theta_{\mathcal{C}}) = \sum_{i \in \mathcal{C}} (\hat{L}_i - L_i)^2, \quad (3)$$

Table 2: Dice scores of the different methods with different parameterizations.

Patch size	No. of edges	CRF-Net	T-NET$^{\#}$	LearnGC$^{\#}$
$7 \times 7 \times 11$	3	0.598 ± 0.078	0.593 ± 0.082	0.640 ± 0.085
	4	0.599 ± 0.081		
	5	0.597 ± 0.081		
$9 \times 9 \times 13$	3	0.599 ± 0.090	0.599 ± 0.089	0.655 ± 0.080
$11 \times 11 \times 15$		0.620 ± 0.081	0.618 ± 0.084	0.665 ± 0.077
$13 \times 13 \times 17$		N/A*	0.639 ± 0.073	0.677 ± 0.070

N/A*: Fail due to limited memory.

Table 3: Summary of the improvements by adapting CRF as a post-processing step on different datasets in Chen et al. (2018).

Dataset	Mean IOU
PASCAL VOC 2012	1.34%
PASCAL-Context	2.00%
PASCAL-Person-Part	0.54%
Cityscapes (VGG-16)	1.05%
Cityscapes (ResNet-101)	0.40%

where label probability L_i is generated from ground truth labels. Note that the number of the connected edges of each node can be different. To consider the neighbor edges of the target node in an end-to-end network, we need to fix the number of connected edges, e.g. we can choose three edges from the neighbors as Fig. 3 shows. The performance of the CRF-Net with different numbers of edges is studied in Section 2.3.2.

2.3. Experiments

2.3.1. Comparison with the proposed LearnGC

To evaluate the performance of the proposed CRF-Net, we compared it with T-NET$^{\#}$ and the proposed LearnGC$^{\#}$. Here, CRF-Net used three neighbor edges; T-NET$^{\#}$ directly used the estimated t-link weight from single scale T-NET to classify scars, and LearnGC$^{\#}$ combined the estimated t-link and n-link weights based on a single-scale network to segment the scars. In this experiment, the patch size was set to $7 \times 7 \times 11$ pixel, and the scale number N_s was set to 1, due to the limited memory. The balancing parameter λ in this study was set to 0.6, and the random shift range $\mathcal{R}$ was set to half of the patch length.

The results are presented in Table 1. Compared to T-NET$^{\#}$, CRF-Net achieved 0.5% improvement. By contrast, LearnGC$^{\#}$, with n-link, improved 4.7% in Dice scores. Note that the contribution of CRF is not evident in Dice scores, but the difference was statistically significant ($p = 0.012$) between CRF-Net and T-NET$^{\#}$. Hence, the neighbor features considered by CRF-Net did improve the segmentation performance. Nevertheless, the proposed CRF-Net was performed and locally optimized on a sub-graph, which limited its performance. In contrast, the proposed LearnGC$^{\#}$ can globally optimize the whole graph, even though the training and classification were disconnected in a non-end-to-end framework.

Table 4: Summary of the improvements by adapting CRF as a post-processing step on different datasets in Kamnitsas et al. (2017).

Dataset	Mean Dice
BRATS 2016	1.1%
ISLES-SISS 2015	2.0%

2.3.2. Parameters studies

We evaluated the performance of CRF-Net with different numbers of edges and patch sizes in this study. The performance was compared with that of T-NET$^{\#}$ and LearnGC$^{\#}$. The results are presented in Table 2.

One can find that the different numbers of edges, from 3 to 5, used by CRF-Net did not significantly affect the classification performance, namely their Dice scores are not statistically different (p>0.2). By contrast, the patch size was more effective. It is worth mentioning that the improvement of n-link using patch size $13 \times 13 \times 17$ was 3.8% when scale number N_s=1 and was 1% when N_s=3. This indicates that the multi-scale patch can include more global information from neighborhood.

2.4. Conclusion

The proposed CRF-Net was locally optimized on a single node due to the memory limitation, as we discussed in Section 2.1. Note that the main contribution of conventional CRF is that it guarantees the global maximum likelihood convergence (Lafferty et al., 2001), compared to the maximum entropy markov model and the other discriminative Markov models based on directed graphical models. A CRF-based deep neural network working on the whole graph is still an open question.

3. Discussion of n-link weight

The improvement by n-link in terms of Dice scores is limited (1.0%). However, the main function of n-link in this work is to reduce the noise and patchy segmentation results. Similarly, the fully connected CRF (FC-CRF) employed in Chen et al. (2018) was used as a post-processing step, instead of end-to-end training, to remove false positives and refine object boundaries, and the FC-CRF in Kamnitsas et al. (2017) was also a post-processing step to refine the output of the segmentation network.

For comparisons, we summarize the improvements by adapting CRF for post-processing from the literature. The

summarized results are presented in Table 3 and Table 4. The improvement of the methods with CRF, compared to the methods without CRF, ranges from 0.4% to 2.0% in terms of mean IOU, as they were reported in Chen et al. (2018), and ranges from 1.1% to 2.0% in terms of mean Dice in Kamnitsas et al. (2017).

In our work, the main function of graph-cuts as a post processing step is to refine the segmentation by smoothing the boundaries. The improvement in terms of mean Dice score, i.e., 1.0%, was marginal and similar to the FC-CRF reported in the literature.

References

Chen, L.C., Papandreou, G., Kokkinos, I., Murphy, K., Yuille, A.L., 2018. Deeplab: Semantic image segmentation with deep convolutional nets, atrous convolution, and fully connected CRFs. IEEE transactions on pattern analysis and machine intelligence 40, 834–848.

Kamnitsas, K., Ledig, C., Newcombe, V.F., Simpson, J.P., Kane, A.D., Menon, D.K., Rueckert, D., Glocker, B., 2017. Efficient multi-scale 3D CNN with fully connected CRF for accurate brain lesion segmentation. Medical image analysis 36, 61–78.

Krähenbühl, P., Koltun, V., 2011. Efficient inference in fully connected crfs with gaussian edge potentials, in: Advances in neural information processing systems, pp. 109–117.

Lafferty, J., McCallum, A., Pereira, F.C., 2001. Conditional random fields: Probabilistic models for segmenting and labeling sequence data .
